# Responders to Cervical Facet Platelet-Rich Plasma Demonstrate Synergistic Improvements in Pain and Isometric Strength in Chronic Whiplash-Associated Disorders: A Series of Mediation Analyses

**DOI:** 10.3390/clinpract15080135

**Published:** 2025-07-23

**Authors:** Ashley D. Smith, Benjamin Andruski, George Deng, Colin Bouma, Marc Pesant, Fiona Magill, Robert Burnham

**Affiliations:** 1 Department of Clinical Neurosciences, Cumming School of Medicine, Calgary, AB T2N 1N4, Canada; benjamin.andruski@ucalgary.ca (B.A.); george.deng@ucalgary.ca (G.D.); 2 Vivo Cura Health, Calgary, AB T2E 2P5, Canada; fiona.magill@mail.utoronto.ca (F.M.); rburnham@capriclinic.ca (R.B.); 3 Department of Anesthesiology, Perioperative and Pain Medicine, Cumming School of Medicine, Calgary, AB T2N 1N4, Canada; 4 Renewal Physiotherapy, Calgary, AB T2H 0L3, Canada; cbouma@cbihealth.ca; 5 Lifemark Health–Crowchild Twin Arena, Calgary, AB T3L 1L4, Canada; marc.pesant@lifemark.ca; 6 Department of Medicine, Faculty of Medicine & Dentistry, University of Alberta, Edmonton, AB T6G 2R7, Canada

**Keywords:** platelet-rich plasma, whiplash-associated disorders, cervical facet joint, rehabilitation, isometric strength, range of motion, neck disability index

## Abstract

Background/Objectives: Platelet-rich plasma (PRP) is emerging as a safe and effective treatment for facet-mediated pain. Studies have demonstrated reductions in pain and improvements in function, both in the short (3 months) and longer term (6 and 12 months). The mechanisms underlying clinical improvements are largely unknown. It is also unclear whether reported outcomes are due to the PRP administered or concurrently applied rehabilitation. Methods: A prospective case series was conducted in a single, multidisciplinary chronic pain centre. Forty-two participants with chronic WAD and cervical facet-mediated pain who received PRP (64% female; mean age (SD) 42.8 (11.6) years; median WAD duration [IQR] 23 [18,29] mths), attended rehabilitation, and reported successful outcomes 3 months post-PRP fulfilled the inclusion criteria. Measures of pain, cervical isometric strength, and range of motion were collected at baseline and 3 months post-PRP. Mediation analyses were performed to determine how these factors influenced disability. Results: Participants demonstrated clinically significant and relevant improvements in pain, disability, and isometric strength measures (all *p* < 0.01). Causative mediation analyses demonstrated independent direct, but not indirect, effects of both pain and strength on disability (both *p* < 0.001), with no direct or indirect effects of cervical ROM on disability.

## 1. Introduction

Platelet-rich plasma (PRP) is emerging as a safe and effective treatment for cervical and lumbar spine facet-mediated pain. Studies have demonstrated reductions in pain and improvements in function, both in the short (3 months) [1] and longer term (6 and 12 months) [2,3,4]. However, it is unclear if the improvements in reported outcomes are solely due to the PRP administered or concurrently applied rehabilitation [1,2].

From a purely PRP perspective, there are two broad factors which may be associated with improved outcomes over time—an anti-inflammatory effect [5] and a tissue healing effect [6]. Platelet-rich plasma results from centrifugation of whole blood, and as the name suggests, contains a concentration of plasma rich in platelets and hence growth factors. Healing may occur through increased cellular metabolic activity while reducing cell apoptosis, increasing blood supply to the injured tissues via angiogenesis, and increasing tensile strength of the new tissue [7]. All of these factors may contribute to improved healing and functional outcomes. However, it is unclear if rehabilitation supplements these effects.

To determine if rehabilitation complemented the effects of PRP, we recently performed a single case experimental design study in people with chronic cervical facet-mediated pain following a whiplash injury [8]. Three randomized participants were prescribed 10 sessions of rehabilitation following peri-articular PRP injection, and three participants were provided with a booklet containing advice and information regarding whiplash injuries and exercises to perform. These participants were also able to contact a physician at any time during the study. Participants recorded daily pain levels for the 6 weeks of treatment, with secondary generalization measures collected at the end of treatment and 3 months post-PRP to further evaluate outcomes. Of interest is that the greatest rate of pain reduction occurred in the days following administration of PRP. This suggested that improvements possibly resulted from anti-inflammatory effects that were initiated upon administration of PRP. However, the data also indicated that participants maintained improvements in the 3 months following the trial and further improvements occurred in people that initially did not attend rehabilitation, but subsequently received rehabilitation following the trial. Thus, this also suggests that tissue healing properties (increased tensile strength of the new tissue) were possibly associated with improved function. The study was underpowered to determine if rehabilitation provided any extra benefit beyond that provided by PRP and usual care. The study also did not measure outcomes associated with rehabilitation, such as range of motion or strength, to determine if these measures improved following PRP with or without rehabilitation.

Thus, the current study aimed to investigate if (a) there was a significant improvement in clinical features, in particular, cervical range of motion and isometric strength following 3 months of rehabilitation, and (b) if these features mediated the improvements in pain and disability. The study sample exclusively focussed on participants who reported a successful outcome following PRP to investigate the maximum possible benefits that could be achieved with rehabilitation.

## 2. Materials and Methods

### 2.1. Design

A prospective case series was performed at a community multidisciplinary chronic pain centre in Calgary, Alberta between October 2019 and December 2022. Patients provided consent for their anonymized data to be initially entered into the registry database. Registry data were extracted on 30 March 2023 for retrospective analysis, which was approved by the Conjoint Health Research Ethics Board (ID#: REB20-0355). Patient consent was waived as anonymized data was extracted from a registry database.

### 2.2. Participants

Participants attended the clinic for further evaluation of persistent neck pain, secondary to a motor vehicle collision, after previously not responding to conservative therapy. Participants completed an electronic intake form and were examined by an experienced physiotherapist (PhD-trained with over 30 years of experience) and medical doctor/specialist (physiatrist). If their history and examination [9] suggested that the facet joints may be responsible for their neck pain, the participant was referred for diagnostic medial branch blocks. Subsequent study enrolment occurred when the participant was selected from an ethics-approved registry database after fulfilment of the following inclusion criteria:

(a) Aged 18 or older.
(b) Presented with a whiplash-associated disorder (WAD) grade II classification (presence of neck complaint and musculoskeletal signs such as reduced cervical ROM and point tenderness).
(c) Received a single autologous injection of ultrasound and fluoroscopically guided PRP into the cervical facet joints and peri-articular joint margins (1) after demonstrating a positive response (greater than 80% relief of index pain or greater than 50% relief of pain AND significant improvement in performing a previously limited activity of daily living) to a single diagnostic medial branch block, as previously reported [1].
(d) Attended rehabilitation (self-reported) in the 3 months post-PRP and a 3-month review, which included repeat patient-reported outcomes and physical measures being collected.
(e) A successful outcome was reported after 3 months, defined as a greater than 15% reduction in pain intensity, which has been demonstrated to be the minimal clinically important change for chronic musculoskeletal pain intensity, when measured on a numerical rating scale, as was instituted in this study [10].

Patients were excluded from the study if they initially presented with concussion symptoms or presented with WAD III (neck complaint and neurological signs) or WAD IV (neck complaint and fracture or dislocation).

### 2.3. Conservative Rehabilitation

Participants were encouraged to attend conservative rehabilitation delivered by a physiotherapist. Participants were counselled that rehabilitation should primarily consist of progressive, goal-directed exercise to actively restore any measurable physical impairments regarding strength, endurance, or cervical mobility deficits. Participants were cautioned against rehabilitation that primarily focussed on the use of passive modalities, inclusive of manual or needle-based therapies provided as unimodal treatments. Participants were also instructed that their cervical mobility and strength would be re-measured 12 weeks post-PRP. Thus, participants were motivated to address physical impairments measured at time of intake, prior to receiving PRP.

### 2.4. Outcomes

Primary Outcome Variable:

Neck disability index (NDI: 0–100%): The NDI is a reliable, validated, Likert-scaled (0–5) 10-item questionnaire that evaluates activities of daily living that may be affected by neck pain [11,12,13]. The relative percentage change in NDI ([NDI_pre_—NDI_post_]/NDI_pre_*100) was calculated and formed the primary dependent variable of interest. Categories for mild (0–28%), moderate (30–48%), and severe (>48%) levels of disability have been established [14]. The minimally clinically important difference (MCID) for mechanical neck disorders has been calculated to be 19% [15].

Predictors:

Pain intensity was defined as the average pain within the prior week, as measured by a numerical rating scale embedded within the Pain, Enjoyment of Life and General Activity (PEG) scale [16]. The relative percentage change in pain ([pain_pre_-pain_post_]/pain_pre_*100) was calculated. The MCID for chronic musculoskeletal pain has been defined as a 15.0% change [10]. Scores of 6–7 correspond to moderate pain in terms of pain-related interference with functioning [17].

Physical Measures:

Cervical range of motion (ROM) was measured using a standard universal generic bubble inclinometer for flexion/extension and side flexions, whilst a universal goniometer (UG; Baseline, Taiwan) was used for rotation. Measurements were performed in a sitting position, with participants maintaining an upright thoracolumbar posture throughout. Measurements were repeated if the participant or assessor determined that thoracolumbar movement occurred. Participants were provided with a ‘warm-up’ and instructed to move through a full available pain-free range. A single measure in each direction was then recorded. Reliability (ICCs ranging from 0.89 to 0.94) has been demonstrated for inclinometry [18] and also for the UG (ICCs greater than 0.80) [19]. The MCID for total cervical ROM is 33^0^ [20].

Cervical isometric strength was measured using a standardized, calibrated digital hand-held dynamometer (MicroFET 2 force gauge, Hoggan Health Industries, Salt Lake City, UT, USA). Participants were seated comfortably on a plinth without back, arm, or feet support and were asked to place their hands in their lap to prevent bracing. All test positions were performed with the neck in neutral. In each “break” test position, the participants were instructed to gradually increase their maximum cervical muscle force over 4 s to meet that of the assessor, maintaining the starting neck position (a “break” test is an eccentric test in which the assessor exceeds the maximum resistance produced by the participant and causes lengthening of the muscle). Participants could stop the test at any point during the assessment and were instructed to stop should any pain arise. A single maximum force generated in Newtons for flexion, extension, and right- and left-side flexion was recorded, and the sum of these measures was calculated and defined as ‘Total Neck Strength’. Test–retest intra-class correlation coefficients (ICCs) have previously demonstrated high reliability, ranging from 0.94 to 0.97 for all tested directions [21]. The same physiotherapist (A.S.) completed all physical measures. He was blinded to responder status at the time of measurements being performed at 3 months.

### 2.5. Data Analysis

**Figure 1:**
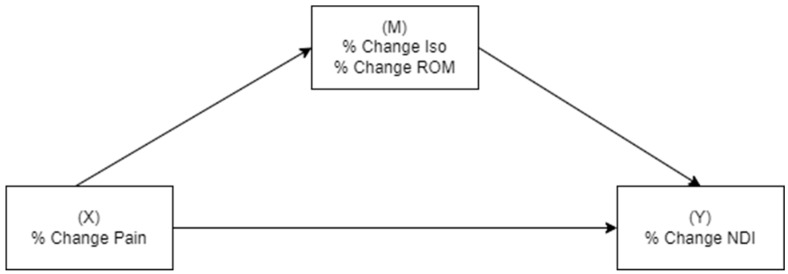
Simple mediation analyses with pain intensity as the independent variable, disability as the dependent variable, and the respective physical measure (isometric strength or cervical range of motion) as the mediator.

**Figure 2:**
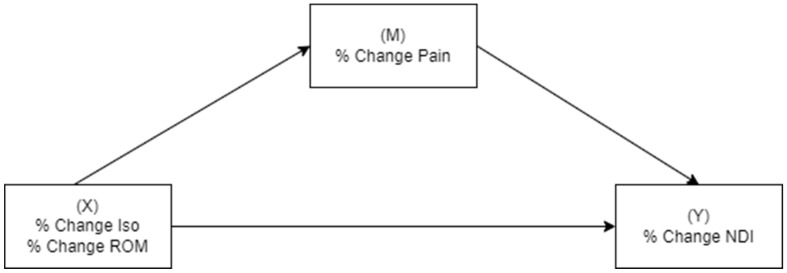
Simple mediation analyses with physical measures (isometric strength or cervical range of motion) as the independent variable, disability as the dependent variable, and pain intensity as the mediator.

Data were analyzed for normality through box plots and Shapiro–Wilk statistics. Paired *t*-tests evaluated changes over time. Zero-order Spearman correlations examined the relationships between pain, physical measures, and disability. A small (0.10–0.29), medium (0.30–0.49), or large effect (0.50–1.00) size was determined according to Cohen’s criteria [22]. The relative percentage change in measures over time was used in mediation analyses, which investigated if physical manifestations (change in total strength and total cervical ROM) mediated the relationship between the change in pain (independent variable) and change in disability (dependent variable) (Figure 1), and conversely if the change in pain mediated the relationship between change in strength/ROM and change in disability (Figure 2). The Preacher and Hayes criteria was used to demonstrate mediation [23]. Firstly, there needed to be an effect to be mediated—an association between percentage change in pain (Model 1) or physical measures (Model 2) and percentage change in disability. Secondly, a significant indirect effect of pain intensity/physical measures on the dependent variable via the tested mediators was required. When criterion 1 was met, the significance of the indirect effect was measured by determining if the 95% confidence intervals did not include zero.

Bootstrapped mediation analysis using SPSS (Version 26) dialogue (the PROCESS Procedure: https://www.processmacro.org/index.html (accessed on 1 July 2025), Version 4) examined total, direct, and indirect effects [24]. Using the SPSS dialogue, mediation analyses were performed, specifying Model 4 with the calculations of a bootstrap confidence interval (CI) with 5000 replications. Percentile bootstrapping was used to derive path coefficients and corresponding 95% confidence intervals for indirect effects. Fritz and MacKinnon have previously demonstrated that a sample size of 36 results in power of 0.8 when there are large effect sizes between the independent variable and mediator(s), and mediator(s) and dependent variable, which was anticipated in this study [25]. All analyses were performed using IBM SPSS for Windows Version 26 (SPSS Inc., Chicago, IL, USA) with significance set at 0.05 (Bonferroni correction *p* < 0.008 for multiple comparisons).

## 3. Results

A total of 93 participants received cervical PRP during the specified study period. Of those, 70 attended rehabilitation, with 42 (64% female; mean age (SD) 42.8 (11.6) yrs; median WAD duration [IQR] 23 [18,29] mths) reporting a successful outcome 3 months post-PRP and attending review for re-evaluation (collection of measures). These formed the study sample under investigation. Of interest, 19 of those who attended rehabilitation reported at least 50% pain relief at 3 months. Participants presented with moderate levels of pain and disability (Table 1). The study sample was selected based on improvements in pain over the 3-month period. Significant reductions in disability and improvements in both isometric strength and ROM were also demonstrated (Table 1).

The most common cervical facet joints injected were C5/6 and C4/5 (Table 2). A substantial number of participants had multiple facet joints injected, with approximately half having bilateral injections (Table 2).

### 3.1. Association Between Pain, Disability, and Physical Measures

There was a significant large positive relationship demonstrated between improvements in pain and disability, isometric strength, and disability (Table 3). Thus, the first requirement for mediation was met. There was no significant association between pain or disability and ROM.

### 3.2. Tests of Mediation

For the first model investigating whether physical measures (isometric strength or ROM) mediated the effect of pain intensity on disability, both models demonstrated a significant direct effect (Table 4). Isometric strength or ROM did not mediate disability. Overall, greater reductions in pain intensity following PRP directly resulted in greater reductions in disability that were independent of physical factors. That is, reductions in pain did not result in improvements in isometric strength or ROM.

For Model 2 (Table 5), there was a direct effect of isometric strength on disability. Pain did not mediate the relationship between isometric strength and disability. These findings support a model in which the influence of improvement in isometric strength over 3 months post-PRP directly reduces disability, independent of reductions in pain.

## 4. Discussion

Participants attending rehabilitation in the immediate 3 months following PRP injection demonstrated clinically significant and relevant improvements in pain, disability, and isometric strength measures. Although improvements in cervical ROM were demonstrated, the clinical relevance of these improvements is unclear. Causative mediation analyses demonstrated independent direct, but not indirect effects of both pain and strength on disability, with no direct or indirect effects of cervical ROM on disability.

Participants in the study demonstrated higher levels of pain and disability than other study samples of chronic WAD, with moderate levels of pain and disability [26,27,28,29]. This is likely due to the tertiary and interventional nature of the clinic where the study was administered. Participants are generally referred for further multidisciplinary evaluation if they do not respond to standard conservative care. Participants demonstrated reduced cervical isometric strength and ROM, when compared to a normative database [29,30], but were not as limited as other WAD cohorts [31,32]. This may have resulted from the fact that our study sample was chosen based on a successful treatment outcome, and thus, people enrolled in the study likely demonstrated improved measures of physical performance at baseline relative to those who did not respond to PRP. This study also differed from most other studies of chronic WAD, in that all participants were homogeneous regarding their anatomical source of pain, having previously reported a positive response to a single diagnostic facet joint injection. Most participants demonstrated multiple areas of cervical facet joint dysfunction, with all but three people responding to multiple levels of diagnostic facet joint injections.

Prior studies investigating PRP delivered to the facet joints have reported significant improvements in pain and disability, both in the short (3 months) and longer (6 and 12 months) term [1,2,3,4]. However, only one of these studies has been controlled [3] and even that study did not report whether participants attended subsequent rehabilitation. In a previous study, involving a single case experimental design, initial daily improvements in pain and disability were identified prior to expected benefits that would have been observed following the 6 weeks of rehabilitation, suggesting that PRP alone was positively effecting change [8]. Given the multiple potential mechanisms associated with PRP, it was speculated that an anti-inflammatory effect was influential in these results. Our current study findings support this idea. There was a direct effect of pain reduction that resulted following PRP which directly influenced disability independent of improvements on strength or ROM. That is, improvements in pain did not result in changes in strength, which then could have resulted in improvements in disability. Instead, pain reduction directly reduced disability, showing that pain reduction is important for people with chronic WAD. Of note is that the standardized regression coefficients were significantly larger in this model, when compared to those demonstrated where strength influences on disability were investigated. This suggests that PRP directly influences pain. As biomarkers were not used in this study, we can only speculate regarding the influence of PRP on pain reduction. Recent research has demonstrated that chronic neck or musculoskeletal pain is associated with inflammation [33,34], and thus, PRP may be modulating these effects. This has been demonstrated in people with knee osteoarthritis [35,36], and requires further research.

Interestingly, our study findings also demonstrated improvements in strength and ROM 3 months post-PRP. However, ROM did not return to the healthy limits demonstrated in other studies [37]. Causative mediation analyses demonstrated that improvements in strength directly resulted in reductions in disability, independent of pain reduction. This supports the result of the SCED, where sustained improvements in pain and disability were reported in those who subsequently pursued rehabilitation post-PRP [8], which suggested that rehabilitation may have influenced outcomes outside of any direct effect of PRP. Thus, strength improvements did not reduce pain, but directly influenced disability. This is an important finding, as it is not always possible to reduce pain, making functional improvement an encouraging outcome. It also suggests that the sprain/strain model of whiplash injury demonstrated in cadavers [38], human volunteers [39], and mathematic modelling [40], which has resulted in capsular ligament sprain [41], nociceptive signalling [42], neuromodulatory [43], inflammatory [44], and electrophysiological [45] changes in animals subject to similar whiplash forces, can be effectively treated. One prior study demonstrated that improvements in pain and disability was possible in people undergoing radiofrequency ablation (RFA) for cervical facet-mediated pain who previously did not respond to substantial conservative therapy [46]. This study expands on those findings (which did not evaluate effects of rehabilitation or measure changes in physical measures). Increased strength after 3 months suggests that the capsule can now tolerate extra load that previously resulted in pain, and with effective pain relief achieved through PRP, patients are able to pursue strengthening to improve their health outcomes.

Irrespective of the underlying mechanisms whereby pain reduction and strength improvements contributed to significant and clinically relevant improvements in disability, these results suggest that the effects of PRP are complemented by a strengthening programme, and the combination of pain relief directly achieved through PRP and strength improvements results in clinically relevant improvements. In fact, isometric strength was restored to normative measures demonstrated for healthy people [30]. Over the last decade, many RCTs providing conservative therapy have only demonstrated modest improvements in disability reduction [26,28,29], suggesting that conservative therapy may have its greatest effect when supplemented by effective pain relief. Previously, only RFA has demonstrated clinically significant reductions in pain and disability over the short to longer term [47,48]. However, RFA requires repeating and is not necessarily a suitable intervention for all people with chronic WAD. As this study demonstrated, whereby most participants had greater than 3 levels of facet joint involvement, PRP may provide sustainable relief where RFA is not indicated [49], or for people who would prefer not to pursue RFA.

This study has several limitations that warrant attention. The study was an uncontrolled case series in responders to cervical facet joint PRP without a control group, which may introduce sample selection bias and hence generalizability to all those receiving PRP. Attendance for conservative rehabilitation was not formally monitored and was self-reported 3 months post-PRP. Even though strength and ROM improvements were demonstrated, it is unclear what features of rehabilitation contributed to these improvements. For those attending physiotherapy at the study clinic, specific attention was placed on improving isometric strength and endurance, with manual therapy generally performed to assist with restoration of cervical ROM. However, not all participants in the study attended the clinic for rehabilitation and adherence to home exercise was not monitored; thus, it is unclear how rehabilitation influenced outcomes. Participants in this study could also afford to attend rehabilitation, which required private funding. Greater socioeconomic advantage may have contributed to improvements demonstrated [50], and thus caution needs to be taken when generalizing to the larger WAD population. Other factors may have also influenced the results. For example, fear has been demonstrated to influence isometric strength [51], which was not measured. Thus, the findings may be confounded by unknown variables that influence strength. The study also has certain strengths. The longitudinal nature of the study of this prospective case series allows participants to act as their own controls. We were also able to use advanced statistics to investigate causation, although given the medium (and not large) relationships between the mediators and dependent variable, the original intended power of 0.8 was not achieved [25]. A decent sample size was available for analysis, although higher numbers would allow investigation of other factors. A randomized controlled clinical trial is recommended to fully evaluate the role of rehabilitation which cannot be fully determined by an uncontrolled study.

In the future, use of biomarkers would assist with determination of the role of inflammation. Studies are required to ascertain who may or may not respond to PRP. In this study, 70% of participants responded to PRP and rehabilitation. Appropriate selection criteria and dose responsiveness of PRP and optimal rehabilitation parameters require further research to optimize treatment outcomes.

## 5. Conclusions

In those responding to cervical spine PRP and physiotherapy, improvements in pain could not be attributed to improvements in physical measures. However, improvements in disability were associated with independent improvements in both pain and strength, suggesting a synergistic effect optimizes rehabilitation outcomes.

## Figures and Tables

**Table 1:** Pain, disability and physical measures of study sample. Mean numeric pain rating scales (NRS), neck disability index (NDI) scores, total cervical (flexion, extension, right- and left-side flexion and right and left rotation) range of motion (ROM), and total (flexion, extension, right- and left-side flexion) isometric strength (Newtons: N) for participants immediately prior to and 3 months after platelet-rich plasma (PRP) injections. CI = confidence interval.

	Pre-PRP	3 Months Post-PRP	95%CI of the Difference	Significance
NRS (/10)	6.5 (±1.8)	3.5 (±2.0)	[−3.6, −2.5]	<0.001
NDI (%)	43 (15)	29 (15)	[−17, −10]	<0.001
ROM (^0^)	276 (54)	302 (55)	[9, 48]	0.006
Iso (N)	180 (100)	258 (123)	[38, 135]	0.001

**Table 2:** Cervical injection characteristics of study sample. The number of people receiving platelet-rich plasma (PRP) injections at each spinal level, the number having multiple levels, and the number receiving unilateral or bilateral PRP.

Level Injected	C2/3	C3/4	C4/5	C5/6	C6/7	C7/T1
Number	19	24	30	32	15	1
Total Levels	1	2	3	4	5	Uni/Bilateral
Number	3	11	17	10	1	22/20

**Table 3:** Zero-order Pearson correlations between percentage changes over time (pre- and 3 months post-PRP injections) for measures of pain (numerical rating scale), disability (neck disability index), isometric strength (Iso), and range of motion (ROM). ** *p* < 0.01.

	% Change NRS	% Change NDI	% Change Iso	% Change ROM
% change NRS	-	0.70 **	0.08	−0.23
% change NDI		-	0.53 **	0.14
% change Iso			-	0.52

**Table 4:** Model 1—summary of direct and indirect effects for models investigating changes over time between pain (independent variable) and disability (dependent variable) with physical measure (isometric strength or range of motion) mediators. Measures are standardized regression coefficients.

Outcome	Mediator	Direct Effect of Pain	Direct Effect	Indirect Effect	Total Effect
Disability	Isometric Strength	***p*** **< 0.001**	**0.83 (0.53, 1.13)**	0.05 (−0.12, 0.32)	**0.88 (0.49, 1.27)**
	Range of Motion	***p*** **< 0.001**	**1.10 (0.70, 1.51)**	−0.10 (−0.22, 0.08)	**1.00 (0.56, 1.44)**

Note: Each analysis involving a single mediator is represented in each row. Bootstrap 95% confidence intervals that do not contain zero are significant at *p* < 0.05 (in bold). Disability = neck disability index.

**Table 5:** Model 2—summary of direct and indirect effects for models investigating changes over time between physical measures (independent variable) and disability (dependent variable) with pain intensity as mediator. Measures are standardized regression coefficients.

IV	Mediator	Direct Effect of IV	Direct Effect	Indirect Effect	Total Effect
Isometric Strength	Pain Intensity	***p*** **< 0.001**	**0.16 (0.08, 0.24)**	0.02 (−0.06, 0.11)	**0.17 (0.05, 0.30)**
Range of Motion	Pain Intensity	***p*** **= 0.03**	**0.48 (0.14, 0.92)**	−0.28 (−1.02, 0.16)	0.20 (−0.53, 0.93)

Note: Each analysis involving a single independent variable (IV) is represented in each row. Bootstrap 95% confidence intervals that do not contain zero are significant at *p* < 0.05 (in bold).; pain intensity = numerical pain rating scale.

## Data Availability

Data are available through contact with the primary author.
